# Association between systemic immune-inflammatory index and systemic inflammatory response index with body mass index in children and adolescents: a population-based study based on the National Health and Nutrition Examination Survey 2017-2020

**DOI:** 10.3389/fendo.2024.1426404

**Published:** 2024-10-31

**Authors:** Lisha Luo, Lin Chen, Jukun Song, Xiuqi Ma, Xike Wang

**Affiliations:** ^1^ Guizhou Medical University, Guizhou, China; ^2^ Shanghai Children’s Medical Center Guizhou Hospital, Shanghai Jiao Tong University School of Medicine, Guizhou, China; ^3^ Affiliated Dental Hospital of Guizhou Medical University, Guizhou, China; ^4^ Guizhou Provincial People’s Hospital, Guizhou, China

**Keywords:** body mass index, inflammation, systemic inflammatory response index, systemic immune inflammation index, pediatric obesity

## Abstract

**Background:**

The Systemic Immune-Inflammatory Index (SII) and Systemic Inflammatory Response Index (SIRI) are novel composite inflammatory markers. Previous studies suggest that obesity in individuals correlates with persistently low levels of chronic inflammation. This study aims to explore the association between SII and SIRI and Body Mass Index (BMI) among children and adolescents.

**Methods:**

A cross-sectional survey was conducted using the National Health and Nutrition Examination Survey (NHANES) dataset from 2 consecutive cycles from 2017-2020. Multivariate linear regression models were employed to examine the linear relationships between BMI and SII and SIRI. Non-linear associations were explored using smooth curve fitting and threshold effect analysis.

**Results:**

A total of 2980 children and adolescents aged 6-19 years were included in this population-based study. In the population description of body mass index categories, we found progressively higher levels of SII and SIRI, notably peaking among obese children (SII mean ± SD: 528.83 ± 285.46; SIRI mean ± SD: 1.12 ± 0.79). Weighted multivariate linear regression confirmed a significant positive association between BMI and both inflammatory indices (*P* < 0.0001). Subgroup analyses revealed consistent correlations across gender divisions and highlighted a non-linear relationship between BMI and SII.

**Conclusions:**

SII and SIRI are positively associated with BMI in children and adolescents, indicating their potential as markers for assessing systemic inflammation in pediatric obesity. Further large-scale prospective studies are required to substantiate these findings.

## 1 Background

Obesity and its associated comorbidities have become significant global health challenges, with obesity now recognized as the fifth leading cause of mortality worldwide. According to the World Health Organization, childhood obesity ranks among the most critical challenges of the 21st century, affecting over 100 million children globally. Obesity in youth impacts nearly all organ systems, predisposing them to cardiovascular diseases, type 2 diabetes (T2D), endocrine disorders, autoimmune diseases, depression, and cognitive impairments, These conditions significantly elevate the risk of premature death. One of the primary causes is obesity-induced chronic inflammation, characterized as a low-grade, persistent inflammatory response by metabolic cells to excess nutrients and energy, termed metabolic inflammation. This condition has endocrine, paracrine, and metabolic implications that heighten the long-term risk of several severe illnesses. (1–3). Body Mass Index (BMI) is a simple index measured by calculating weight (kg)/height (m^2^), which helps determine whether a child is overweight or obese because of its close correlation with the amount of body fat, and is widely used as a measure of obesity and obesity prevalence (4, 5), Higher Body Mass Index (BMI) and its increase during childhood and adolescence correlate with a rise in adult and central obesity, it is also an important preventable cause of death and is a major cause of cardiovascular diseases such as heart failure and stroke, as well as other serious diseases stroke and cardiovascular diseases, skeletal system disorders and malignant tumors, are major risk factors (6). Current studies have demonstrated that neutrophils, lymphocytes, platelet count, neutrophil-to-lymphocyte ratio (NLR), platelet-to-lymphocyte ratio (PLR) and systemic immune inflammation index (SII) are associated with BMI in adults, but there is insufficient information about the use of these markers in childhood obesity (7).

CBC-derived inflammatory biomarkers such as NLR, PLR, inflammatory response index (SIRI) and SII are associated with BMI in adults. SIRI and SII are used as prognostic factors for various diseases (8). unlike traditional inflammation indices such as NLR and PLR, the systemic immune inflammation index (SII) and systemic Inflammatory Response Index (SIRI) are new composite indices that contain three separate leukocyte subpopulations and platelets, reflecting the interplay of thrombocytosis, inflammation, and immunity. A study by Hu et al. (9) demonstrated that the SII reflects the local immune response and the level of systemic inflammation across the entire human body; whereas, the SIRI is a more comprehensive inflammation based on the count of monocytes, neutrophils, and lymphocytes marker (10), which is a good indicator of the chronic inflammatory state of the body (11).

There are few studies on the correlation of BMI with SII and SIRI, so this study used data from a large sample of people aged 6 to 19 years from the National Health and Nutrition Examination Survey (NHANES) to investigate the association between SII and SIRI and BMI in children and adolescents.

## 2 Methods

### 2.1 Study population

**Figure 1:**
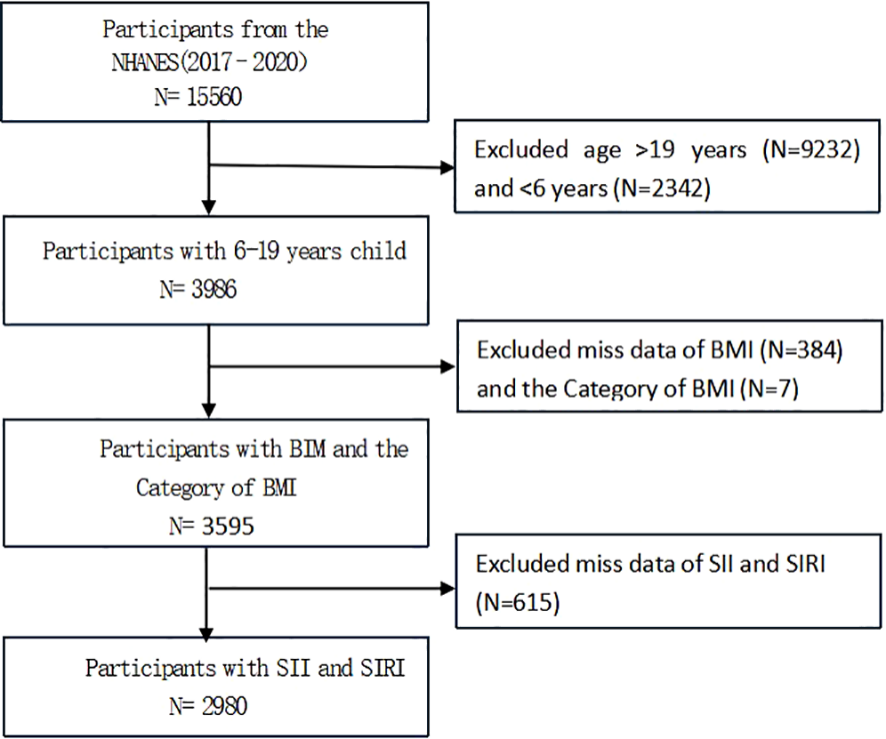
Flowchart of participant selection.

NHANES is a representative U.S. national population survey that uses complex, multistage, and probability sampling methods to provide extensive information about the nutrition and health of the general U.S. population (12). The current survey used the US NHANES dataset from 2 consecutive cycles from 2017-2020, and excluded 9,232 participants aged >19 years, 2,342 participants aged <6 years, 384 participants with missing data on BMI and 7 with missing data on BMI categories, and 615 participants with missing data on SII and SIRI from the 15,560 eligible individuals; the study ultimately included 2980 participants; **Figure 1** illustrates the sample selection flowchart.

### 2.2 Assessment of BMI

BMI was designed as the exposure variable. Body Mass Index (BMI) was calculated as weight in kilograms divided by height in meters squared. Cutoff criteria are based on the Centers for Disease Control (CDC) growth chart “BMI-for-age charts, 2 to 20 years, by sex and age” from the National Health and Nutrition Examination Survey; Age in months was used to match age in months from BMI growth chart data, separately for males and females. There are four categories: 1. Underweight (BMI<5th percentile); 2. Normal weight (BMI 5th to 85th percentiles); 3. Overweight (BMI 85th to 95th percentiles); 4. Obese (BMI≥95th percentile).

### 2.3 Assessment of SII、SIRI

In this study, the systemic immunoinflammatory index (SII) and systemic inflammatory response index (SIRI) were used as outcome variables, where SII was derived from platelet count × neutrophil count/lymphocyte count (13), SIRI was derived from monocyte count × neutrophil count/lymphocyte count (14), and all calculations were expressed as ×10^3^ cells/μl. Lymphocyte, neutrophil, monocyte, and platelet counts were performed using a fully automated hematology analyzer (Kurt^®^ Dx H 800 analyzer) for complete blood counts.

### 2.4 Covariates

Covariates in this study included age, gender, race, family poverty to income Ratio (Family PIR), arm circumference, waist circumference, waist-to-height ratio (WHtR), activity level (number of days of activity up to 60 min per day in 7 days), total daily energy intake and fat intake, HDL-Cholesterol, total cholesterol, ultrasensitive C-reactive protein, NLR (neutrophil-to-lymphocyte ratio), PLR (platelet-to-lymphocyte ratio), and further stratified analyses for age, gender and WHtR, with age divided into school-age (6-9 years) and adolescence (10-19 years), in which outweighs and equals 0.5 was defined as centripetal obesity, and less than 0.5 was defined as non-centripetal obesity.

### 2.5 Statistical analyses

This paper was statistically analyzed using EmpowerStats (version 4.0) and R software. Baseline tables of the study population were statistically described by BMI categories; Continuous variables were described by means with standard deviation (SD) and weighted linear regression models. Multiple linear regression analyses were conducted between BMI and SII and SIRI, β-values and 95% confidence intervals were calculated. Three models were used for multivariate testing: model 1 was unadjusted for variables; model 2 was adjusted for gender, age, and race; and model 3 was adjusted for all covariates in the paper. A threshold effect analysis model was used to investigate the relationship and inflection points between BMI and SII and SIRI after adjusting for variables, followed by smoothed curve fitting. It was determined that *P* < 0.05 was statistically significant.

## 3 Results

### 3.1 Baseline characteristics

A total of 2980 children and adolescents aged 6-19 years were included in this study based on inclusion and exclusion criteria, and the mean age of the participants was 12.37 ± 3.92 years. Among these participants, 51.31% were male, 48.69% were female, 16.71% were Mexican American, 9.53% were Hispanic, 29.87% were non-Hispanic white, 25.20% were non-Hispanic black, and 18.69% were of other races. The mean ± standard deviation of SII and SIRI were 450.12 ± 287.14 (10^3^ cells/μl) and 0.94 ± 0.83 (10^3^ cells/μl).

**Table 1** presents all the clinical characteristics of all participants in the form of BIM categories as a column-stratified variable. There were no statistically significant differences in age, total energy, adiposity, and PNR between BMI categories. Comparing to the normal BMI group, the obese group had higher levels of height, weight, WHtR, Arm circumference; Waist circumference. Total-Cholesterol, CRP, SII, SIRI, NLR and lower levels of HDL-Cholesterol; Ratio of family income to poverty. SII and SIRI tended to be higher in the underweight to obese group.

**Table 1:** Weighted characteristics of the study population based on BMI categories.

	Total	Underweight	Normal weight	Overweight	Obese	*P*-value
	N =2980	N =88	N =1672	N =494	N =726	
**Age (years)**	12.37 ± 3.92	12.91 ± 4.33	12.21 ± 3.97	12.54 ± 3.85	12.57 ± 3.79	0.061
**Age staging(%)**						0.005
**School-age period**	858 (28.79%)	25 (28.41%)	525 (31.40%)	126 (25.51%)	182 (25.07%)	
**Adolescence period**	2122 (71.21%)	63 (71.59%)	1147 (68.60%)	368 (74.49%)	544 (74.93%)	
**Gender(%)**						0.013
**Male**	1529 (51.31%)	51 (57.95%)	891 (53.29%)	227 (45.95%)	360 (49.59%)	
**Female**	1451 (48.69%)	37 (42.05%)	781 (46.71%)	267 (54.05%)	366 (50.41%)	
**Weight(kg)**	54.11 ± 24.34	35.88 ± 12.68	44.45 ± 16.54	57.64 ± 19.09	76.15 ± 28.04	<0.001
**Standing Height(cm)**	151.92± 18.54	150.72± 20.25	150.24± 19.33	153.44± 18.00	154.92 ± 16.28	<0.001
**Arm circumference (cm)**	26.34 ± 6.12	20.26 ± 3.47	23.50 ± 4.19	27.76 ± 4.41	32.63 ± 5.89	<0.001
**Waist circumference (cm)**	75.77 ± 16.92	59.88 ± 7.68	67.17 ± 9.67	79.11 ± 10.94	95.23 ± 16.85	<0.001
**WHtR**	0.50 ± 0.09	0.40 ± 0.04	0.45 ± 0.04	0.52 ± 0.04	0.61 ± 0.07	<0.001
**WHtR categories (%)**						<0.001
**≥0.5**	1163 (39.03%)	2 (2.27%)	145 (8.67%)	315 (63.77%)	701 (96.56%)	
**<0.5**	1817 (60.97%)	86 (97.73%)	1527 (91.33%)	179 (36.23%)	25 (3.44%)	
**Race/Ethnicity (%)**						<0.001
**Mexican American**	498 (16.71%)	10 (11.36%)	233 (13.94%)	97 (19.64%)	158 (21.76%)	
**Hispanic**	284 (9.53%)	10 (11.36%)	154 (9.21%)	48 (9.72%)	72 (9.92%)	
**Non-Hispanic White**	890 (29.87%)	23 (26.14%)	520 (31.10%)	159 (32.19%)	188 (25.90%)	
**Non- Hispanic Black**	751 (25.20%)	13 (14.77%)	414 (24.76%)	110 (22.27%)	214 (29.48%)	
**Other Race**	557 (18.69%)	32 (36.36%)	351 (20.99%)	80 (16.19%)	94 (12.95%)	
**Family PIR,(%)**	2.14 ± 1.51	2.43 ± 1.53	2.24 ± 1.56	2.21 ± 1.54	1.82 ± 1.31	<0.001
**Activity level(%)**						0.017
**0-2**	572 (19.19%)	19 (21.59%)	297 (17.76%)	95 (19.23%)	161 (22.18%)	
**3-7**	2011 (67.48%)	49 (55.68%)	1162 (69.50%)	332 (67.21%)	468 (64.46%)	
**-**	397 (13.32%)	20 (22.73%)	213 (12.74%)	67 (13.56%)	97 (13.36%)	
**Energy (kcal)**	2015.79± 865.73	2060.82± 853.08	2035.15 ± 871.68	1963.93± 833.45	2001.03 ± 874.91	0.383
**fat (gm)**	81.35 ± 41.38	81.82 ± 39.87	81.59 ± 41.57	79.05 ± 40.59	82.30 ± 41.67	0.579
**HDL-Cholesterol (mmol/L)**	1.38 ± 0.31	1.53 ± 0.28	1.46 ± 0.30	1.33 ± 0.27	1.20 ± 0.26	<0.001
**Total-Cholesterol (mmol/L)**	4.02 ± 0.72	3.96 ± 0.61	3.97 ± 0.69	4.04 ± 0.77	4.11 ± 0.76	<0.001
**HS.CRP(mg/l)**	1.83 ± 4.37	1.55 ± 5.87	1.12 ± 2.92	1.63 ± 4.35	3.64 ± 6.12	<0.001
CBC count, 103/μl						
**White blood cell**	7.06 ± 2.11	7.13 ± 2.21	6.67 ± 1.92	7.11 ± 1.96	7.94 ± 2.32	<0.001
**Neutrophils**	3.72 ± 1.64	3.59 ± 1.75	3.42 ± 1.51	3.78 ± 1.53	4.39 ± 1.78	<0.001
**Monocyte**	0.56 ± 0.18	0.55 ± 0.17	0.54 ± 0.18	0.56 ± 0.16	0.62 ± 0.19	<0.001
**Lymphocyte**	2.52 ± 0.79	2.72 ± 0.93	2.44 ± 0.76	2.52 ± 0.77	2.66 ± 0.81	<0.001
**Platelet**	282.51 ± 64.28	275.99 ± 80.26	275.06 ± 60.34	281.55 ± 65.87	301.13 ± 66.19	<0.001
CBC-derived indicators						
**SII**	450.12 ± 287.14	396.48 ± 264.10	417.3 ± 276.43	454.90 ± 306.23	528.83 ± 285.46	<0.001
**SIRI**	0.94 ± 0.83	0.85 ± 0.76	0.87 ± 0.84	0.94 ± 0.80	1.12 ± 0.79	<0.001
**NLR**	1.60 ± 1.02	1.43 ± 0.85	1.53 ± 1.03	1.63 ± 1.19	1.75 ± 0.86	<0.001
**PLR**	121.21 ± 43.09	109.20 ± 38.45	121.82 ± 43.71	120.30 ± 42.63	121.88 ± 42.33	0.056

### 3.2 Relationship between BMI and systemic immunoinflammatory index

**Table 2** shows the results of the multivariate regression analyses. In the unadjusted model, SII was highly correlated with BMI [*P* < 0.0001 (7.86, 11.15)]. After adjusting for gender, age, and race variables, the positive correlation remained significant in Model 2 [*P* < 0.0001 (7.52, 11.31)]. After adjusting for all covariates listed in this study, the statistically significant association between SII and BMI remained in Model 3 [*P* < 0.0001 (2.96, 7.25)].

**Table 2:** The association between BMI and SII.

	Model 1 β (95% CI) P-value	Model 2 β (95% CI) P-value	Model 3 β (95% CI) P-value
BMI	9.50 (7.86, 11.15) <0.0001	9.41 (7.52, 11.31) <0.0001	5.10 (2.96, 7.25) <0.0001
Stratified by category of BMI			
**Normal weight**	Reference	Reference	Reference
**Underweight**	30.83 (-26.93, 88.59) 0.2955	38.49 (-18.62, 95.60) 0.1866	-18.11 (-38.63, 2.42) 0.0840
**Overweight**	56.21 (-5.33, 117.76) 0.0735	60.39 (-0.57, 121.34) 0.0523	-18.04 (-42.27, 6.19) 0.1446
**Obese**	145.41 (85.13, 205.70) <0.0001	152.94 (93.12, 212.75) <0.0001	1.65 (-26.96, 30.27) 0.9099
**P for trend**	<0.001	<0.001	0.173
Stratified by gender			
**Men**	7.47 (4.81, 10.12) <0.0001	9.55 (6.52, 12.57) <0.0001	6.42 (2.79, 10.05) 0.0005
**Women**	11.05 (9.07, 13.03) <0.0001	8.79 (6.49, 11.09) <0.0001	3.32 (0.54, 6.10) 0.0195
Stratified by WHtR			
**>=0.5**	7.97 (5.31, 10.63) <0.0001	9.50 (6.21, 12.79) <0.0001	7.02 (3.94, 10.10) <0.0001
**<0.5**	8.91 (5.12, 12.71) <0.0001	6.94 (2.00, 11.88) 0.0059	5.26 (2.33, 8.20) 0.0005
Stratified by age			
**School-age period**	11.56 (5.08, 18.04) 0.0005	11.60 (5.07, 18.12) 0.0005	4.23 (-0.03, 8.50) 0.0522
**Adolescence period**	9.66 (7.91, 11.42) <0.0001	9.81 (8.06, 11.55) <0.0001	4.69 (2.09, 7.29) 0.0004

**Figure 2:**
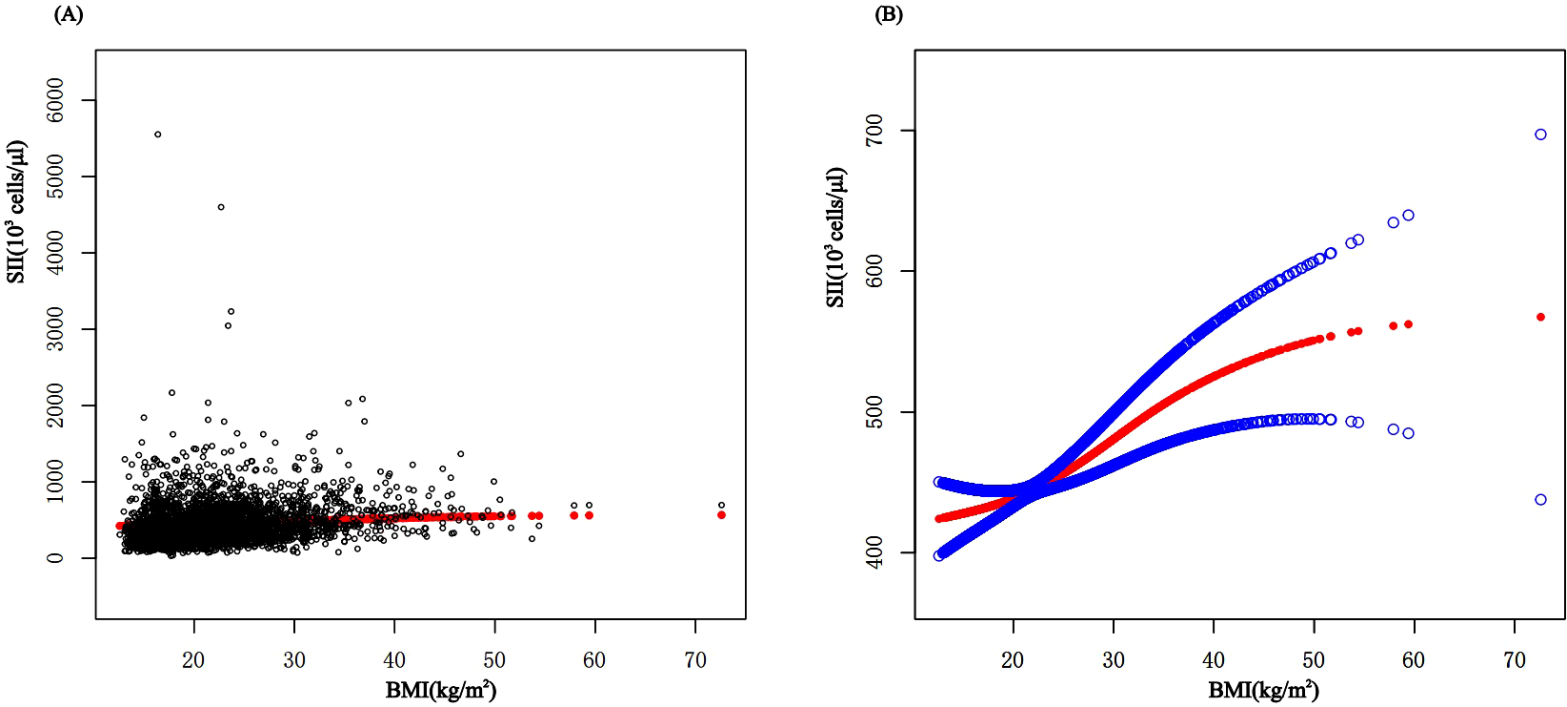
The association between BMI and SII. **(A)** Each black point represents a sample. **(B)** The solid red line represents the smooth curve fit between variables. Blue bands represent the 95% confidence interval from the fit. BMI, Body Mass Index; SII, systemic immune-inflammation index.

**Figure 3:**
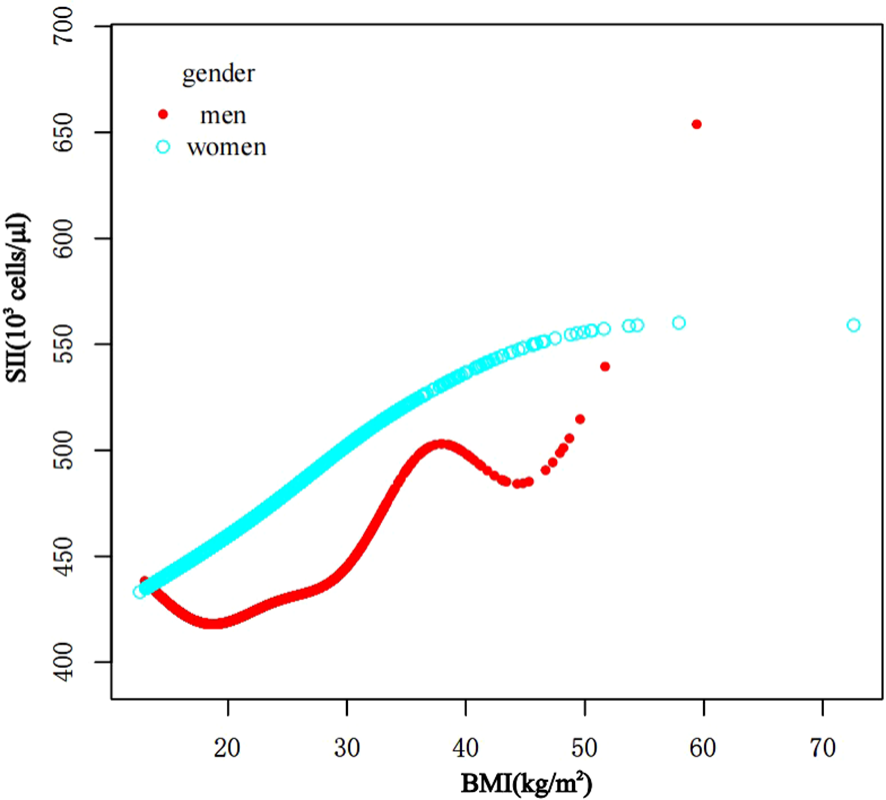
The association between BMI and SII stratified by gender. BMI, Body Mass Index; SII, systemic immune-inflammation index.

In subgroup analyses stratified by gender and waist-to-height ratio (WHtR), the positive correlation between BMI and SII was significantly positive in all stratified analyses, whereas subgroup analyses stratified by age indicated that the positive correlation between BMI and SII was independently significantly positive in the Adolescence period [*P*=0.0004 (2.09, 7.29)], but not in the School-age period was not statistically significant in the 3-model. Using the normal weight group as the reference, SII was strongly positively correlated with the obese group in the unadjusted and partially adjusted model. Smoothed curve fitting was performed to describe the nonlinear relationship between BMI and SII (**Figure 2**). An inflection point between BMI and SII was found using two-stage linear regression model with an inflection point of 48.2 (kg/m^2^). When analyzed stratified by gender, two inflection points were found in males at 16.4 (kg/m^2^) and 41.1 (kg/m^2^) (**Table 3** and **Figure 3**).

**Table 3:** Threshold effect analysis of SII on BMI using two-piecewise linear regression model.

SII	Adjusted b (95% CI) *P*-value
**Inflection point**	48.2
**BMI<48.2**	-181.93 (-321.18, -42.67) 0.0626
**BMI>48.2**	26.10 (4.00, 48.21) 0.0816
**Log likelihood ratio**	<0.001
Men	
**Inflection point**	16.4
**BMI<16.4**	-9.67 (-21.42, 2.08) 0.1069
**BMI>16.4**	6.62 (3.00, 10.24) 0.0004
**Log likelihood ratio**	0.004
**Inflection point**	41.1
**BMI<41.1**	254.33 (209.27,299.39) 0.0574
**BMI>41.1**	74.15 (65.84, 82.47) 0.0364
**Log likelihood ratio**	<0.001

age, gender, race, family poverty to income Ratio, arm circumference, waist circumference, waist-to-height ratio (WHtR), activity level, total daily energy intake and adiposity, HDL-Cholesterol, total cholesterol, ultrasensitive C-reactive protein, neutrophil-to-lymphocyte ratio(NLR), platelet- to-lymphocyte ratio(PLR) were adjusted. SII, systemic inflammatory-immune index.

### 3.3 Relationship between BMI and systemic inflammatory response index

**Table 4** shows the results of the multivariate regression analyses. There was a strong positive correlation between SIRI and BMI in the unadjusted and partially adjusted models (*P*<0.0001). However, there was no statistically significant association between SIRI and BMI in model 3 adjusted for all covariates.

**Table 4:** The association between BMI and SIRI.

	Model 1 β (95% CI) *P*-value	Model 2 β (95% CI) *P*-value	Model 3 β (95% CI) *P*-value
**BMI**	0.02 (0.02, 0.03) <0.0001	0.02 (0.02, 0.03) <0.0001	0.00 (-0.01, 0.01) 0.8888
Stratified by Category of BMI			
**Normal weight**	Reference	Reference	Reference
**underweight**	-0.02 (-0.20, 0.15) 0.8112	0.00 (-0.17, 0.18) 0.9927	-0.03 (-0.11, 0.04) 0.4024
**Overweight**	0.03 (-0.16, 0.22) 0.7670	0.04 (-0.14, 0.23) 0.6419	-0.05 (-0.14, 0.04) 0.2861
**Obese**	0.27 (0.08, 0.45) 0.0042	0.29 (0.11, 0.47) 0.0018	-0.00 (-0.11, 0.10) 0.9695
**P for trend**	<0.001	<0.001	0.711
Stratified by gender			
**Men**	0.03 (0.02, 0.04) <0.0001	0.03 (0.02, 0.04) <0.0001	0.00 (-0.01, 0.02) 0.7616
**Women**	0.02 (0.02, 0.03) <0.0001	0.02 (0.01, 0.02) <0.0001	0.00 (-0.00, 0.01) 0.3968
Stratified by WHtR			
**>=0.5**	0.02 (0.01, 0.03) 0.0003	0.02 (0.01, 0.03) 0.0029	-0.00 (-0.01, 0.01) 0.9408
**<0.5**	0.03 (0.02, 0.04) <0.0001	0.01 (-0.00, 0.02) 0.0724	0.00 (-0.01, 0.01) 0.3655
Stratified by age			
**School-age period**	0.03 (0.01, 0.05) 0.0013	0.03 (0.01, 0.05) 0.0007	0.01 (-0.01, 0.02) 0.3840
**Adolescence period**	0.02 (0.02, 0.03) <0.0001	0.03 (0.02, 0.03) <0.0001	-0.00 (-0.01, 0.01) 0.7891

**Figure 4:**
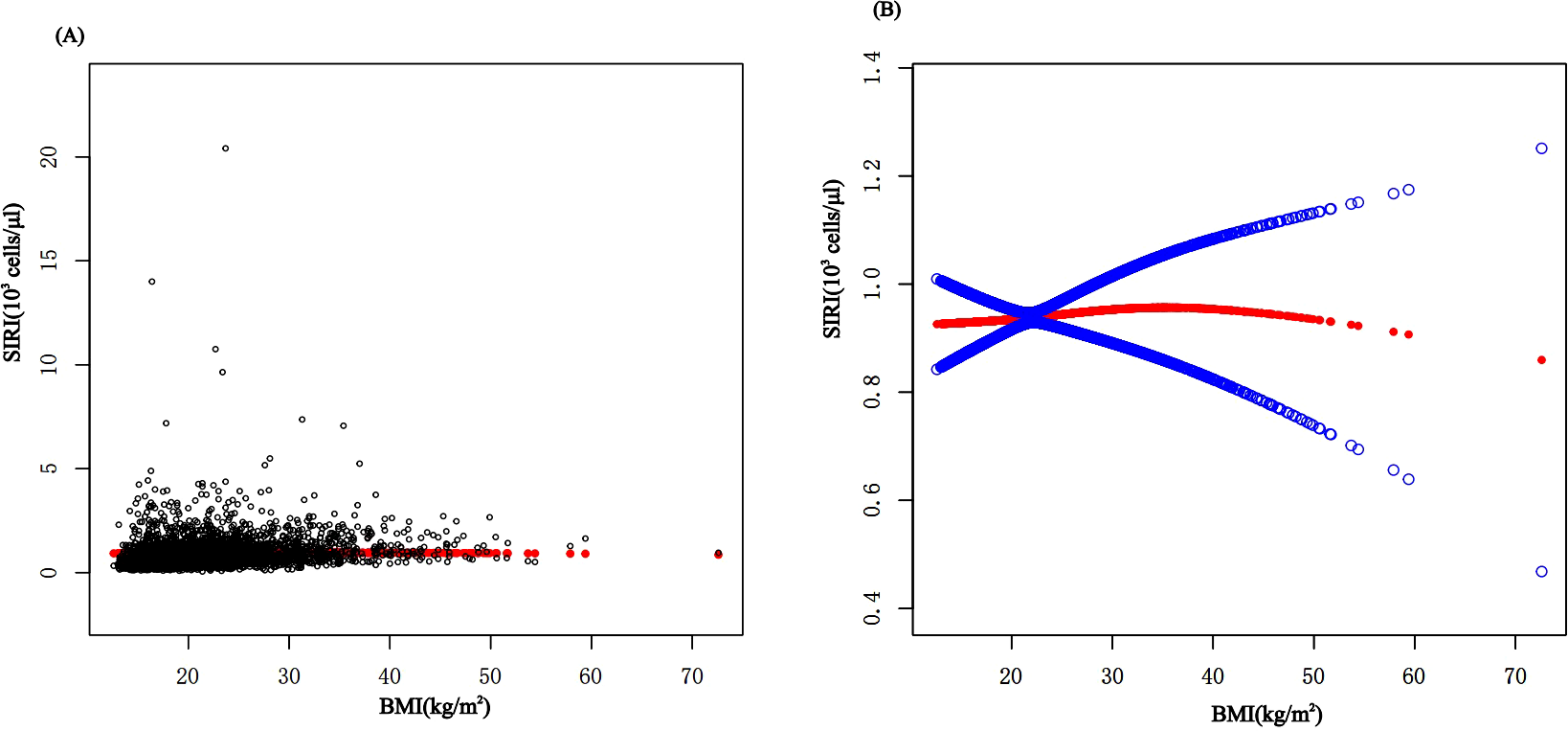
The association between BMI and SIRI. **(A)** Each black point represents a sample. **(B)** The solid red line represents the smooth curve fit between variables. Blue bands represent the 95% confidence interval from the fit. BMI, Body Mass Index; SIRI, system inflammatory response index.

**Figure 5:**
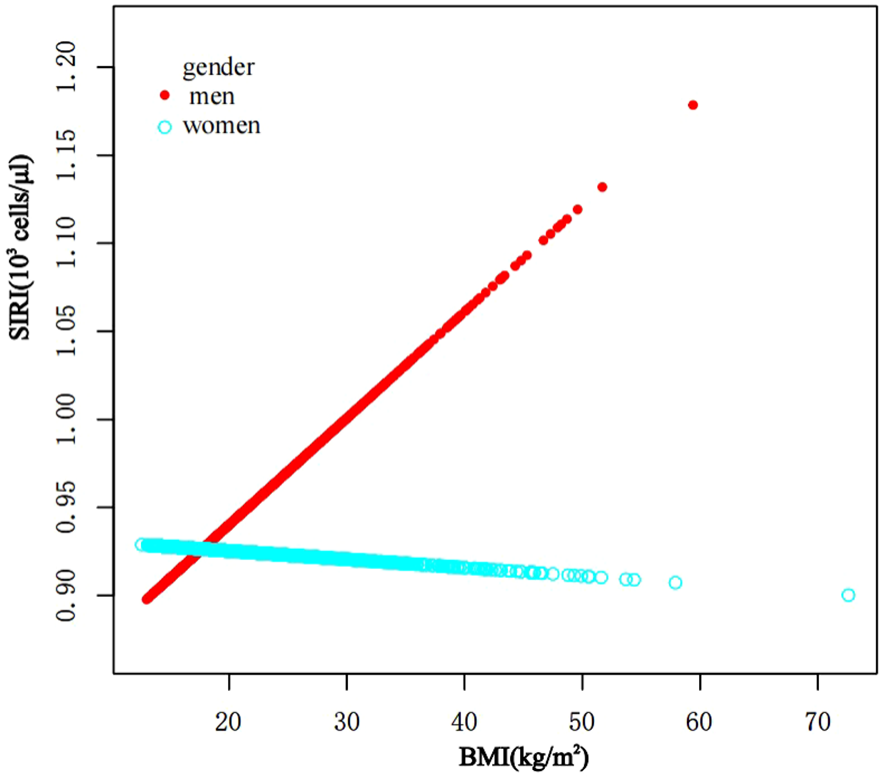
The association between BMI and SIRI stratified by gender. BMI, Body Mass Index; SIRI, system inflammatory response index.

In subgroup analyses stratified by gender, age, and WHtR, the results indicated that the positive correlation between BMI and SIRI was significantly positive in all stratified analyses in unadjusted and partially adjusted models. Smooth curve fitting was used to describe the nonlinear relationship between BMI and SIRI (**Figure 4**). Using two-stage linear regression model we found an inverted U-shaped relationship between BMI and SIRI with an inflection point of 33.3 (kg/m^2^) (**Table 5** and **Figure 5**).

**Table 5:** Threshold effect analysis of SIRI on BMI using two-piecewise linear regression model.

BMI	Adjusted β (95% CI) *P*-value
**Inflection point**	33.3
**BMI<33.3**	0.01 (-0.00, 0.02) 0.1349
**BMI>33.3**	-0.01 (-0.02, 0.00) 0.1794
**Log likelihood ratio**	0.009

age, gender, race, family poverty to income Ratio, arm circumference, waist circumference, waist-to-height ratio (WHtR), activity level, total daily energy intake and adiposity, HDL-Cholesterol, total cholesterol, ultrasensitive C-reactive protein, neutrophil-to-lymphocyte ratio(NLR), platelet- to-lymphocyte ratio(PLR) were adjusted. SIRI, systemic inflammatory response index.

## 4 Discussion

It has been discovered that there is positive correlation between increasing BMI levels and increasing SII and SIRI levels, This relationship is even more significant in obese children, and it is worth noting that stratified analyses revealed variability in this relationship in gender, obesity category, and age period. In the fitted curves of BMI and SII, we found that the curves flattened as BMI increased to 48.2 (kg/m^2^), suggesting that there may be a peak in SII. In the gender subgroup analyses, there were inflection points of 16.4 (kg/m^2^) and 41.1 (kg/m^2^) in male children, There was a positive correlation between BMI and SIRI, while there was an inverted U-shaped relationship between BMI and SIRI with an inflection point of 33.3 (kg/m^2^), suggesting that when the BMI is less than 33.3 (kg/m^2^), the increase in BMI is a risk factor for the increase in SIRI. But the mechanism of which is unclear.

Metabolic inflammation, the term used to describe obesity-induced inflammation, is a chronic, low-grade inflammatory response that is triggered by excess nutrients in metabolic cells. It has been shown that inflammatory signaling carried out by the metabolic cells ultimately causes obesity activation and that levels of circulating cytokines and acute temporal reactants correlate with these levels. Unlike the classical inflammatory response, adipose tissue is the main organ of control of this inflammation (15). While there is a persistent energy surplus, adipocytes compensate for the excess by multiplying and hypertrophying, which results in obesity and an increase in body mass index (BMI). In the case of obesity, there is initially homeostatic stress induced by a positive energy balance and an overall high anabolic state, particularly in adipocytes. which leads to an adaptive inflammatory response through the release of chemokines that permit a healthy expansion of adipocytes with a concurrent reduction in energy stores. In general, when the pressure from the adipocytes reaches a certain point owing to restricted cell and tissue expansion that is insufficient to support additional anabolic pressure, these cells will eventually reach a threshold that triggers an inflammatory process to initiate (16, 17). Inflammatory substances of intestinal origin, food items, or metabolites are among the substances that might trigger inflammation in adipose tissue. Alternatively, the rapid expansion of adipose tissue in obesity may produce internal signals that can lead to inflammation, which includes adipocyte cell death, hypoxia, and mechanical transduction brought on by cell interactions with the extracellular matrix (ECM) (18).

A greater amount of circulating classical CD14 ^+ +^ CD16 ^-^ and intermediate CD14 ^+ +^ CD16 ^+^ monocytes is associated with childhood obesity. These monocytes appear to be important in the development and progression of atherosclerosis and to be a key link between obesity and cardiovascular disease. Furthermore, childhood obesity is accompanied by altered obesity-associated gene expression of monocytes and increased complexity of adult coronary atherosclerosis (19). Prabhakara R et al (20) found that in obesity, the inflammatory mediator IL-1 β reaches the bone marrow through IL-1R and stimulates the growth of hematopoietic progenitor cells *in vitro* through experiments measuring the proliferation of BM progenitor cells in a mouse model, resulting in the production of more monocytes. Additionally, it was discovered that obesity has a feed-forward mechanism wherein inflammatory adipose tissue stimulates the production of more monocytes, resulting in increased inflammation and related disease processes. It is now demonstrated that insulin resistance, pro-inflammatory macrophage accumulation, and adipose tissue inflammation are dependent on CCR2-dependent monocyte recruitment (21). Jongkil Kim et al (22) shown that the involvement of the MCP-1 (monocyte chemotactic protein-1) receptor and CCR2, which are expressed on peripheral monocytes/macrophages, can cause macrophage recruitment to obese adipose tissue (AT). This is one of the main features of adverse metabolic outcomes in dietary obesity. It has been demonstrated that macrophages and other immune cells are the primary inflammatory cells in obesity, releasing the majority of the inflammatory chemicals in the adipose tissue of obese humans and animals (23), such that one of the characteristics of obesity-associated adipose tissue inflammation is an increase in macrophages (24).

Platelets have been shown to play a major role in the inflammatory process and platelet activation is a common feature of inflammatory diseases. Obesity correlates with platelet activation and count. IL-6 is likely to be the main driver, acting synergistically with other interleukins to increase thrombopoietin, of which visceral adipose tissue itself may be an additional source of thrombopoietin, which in turn stimulates megakaryocyte production, leading to thrombocytosis. Mean platelet volume (MPV) is a parameter that reflects platelet activation *in vivo*. In obese patients MPV levels are increased and BMI and MPV are positively correlated and reversible after weight loss. A recent study on the formation of dinoprost analogues *in vivo* found that obese patients had elevated levels of MPV and that body mass index was positively correlated with MPV. In addition, the study found that in patients with abdominal obesity, low-grade inflammation may trigger TX-dependent platelet activation through enhanced lipid peroxidation (25, 26). On the contrary, Y. FURUNCUOğLU et al (6) discovered a significant association between BMI and SII, neutrophil, lymphocyte, and leukocyte counts, as well as all platelet indices (MPV excluded). The current study suggests that altered platelet transcriptome in obese patients as well as obesity-induced changes in plasma lipid composition can lead to increased platelet activation (27).

Neutrophils are the most abundant white blood cells in the blood and are the first immune cells to infiltrate in adipose tissue. Neutrophils are activated and release inflammatory factors that recruit macrophages and other immune cells, including B cells, T cells and NK cells. These immune cells in turn perpetuate the inflammatory state by producing cytokines and chemokines that can reach other parts of the body, creating a systemic inflammatory state. In obese patients neutrophils are increased and there is a significant correlation between the level of neutrophil counts and higher BMI, with overweight individuals with neutrophilia exhibiting higher serum C-reactive protein (CRP) concentrations and larger waist circumferences (28). Obese individuals are able to produce higher levels of inflammatory mediators such as TNF - α, IL - 1 β, IL - 6 and IL - 8, which increase bone marrow granulopoiesis and release neutrophils from the bone marrow into the peripheral circulation. In addition, these inflammatory mediators induce neutrophil detachment from the endothelial wall, leading to neutrophilia. High levels of leptin are observed in obesity, leptin significantly stimulates the emergence of granulocyte macrophage colonies and monocyte and granulocyte precursors, and plasma leptin concentrations significantly correlate with leukocyte counts in overweight and obese individuals, leptin additionally stimulates oxidative bursts of neutrophils, induces chemotaxis, and inhibits apoptosis of these cells, obese adipose tissue is responsible for the promotion of systemic inflammation, which leads to neutrophil production, increased numbers and activation (25, 28, 29). Raghavan et al (30) studied hematological indices in obese women and found a progressive increase in absolute neutrophil counts and neutrophil sorting counts with increasing BMI.

SIRI and SII encompass neutrophil and lymphocyte counts, their discriminative attribute resides in including additional cell types for analysis, Specifically, SIRI considers monocyte counts. whereas SII focuses on platelet counts (31). neutrophil-to-lymphocyte ratio (NLR) is a simple and readily available biomarker and NLR appears to recognize persistent systemic inflammation in overweight individuals. NLR has been found to be significantly higher in obese individuals than in healthy lean individuals and the higher NLR in overweight individuals may reflect the subclinical inflammation that already exists in this population (28). Oliver et al (32) found that lymphocyte sorting counts declined progressively with increasing BMI, and that the absolute lymphocyte counts were offset by a greater increase in neutrophils. In conclusion, elevated levels of neutrophils, lymphocytes, platelets, and monocytes were observed in obese children, with elevated neutrophils offsetting elevated absolute lymphocyte counts. This resulted in elevated levels of SII and SIRI.

## 5 Strengths and limitations

There are certain advantages to this study. firstly, this study is particularly representative of the child and adolescent population since we used a large nationally representative sample from NHANES sources. Second, this study performed subgroup analyses of different variables. Third, there are more studies on the inflammatory mechanisms of obesity and fewer studies on the associated inflammation levels, and to the best of our knowledge this paper is the first to investigate the indicators of obesity inflammation levels.

There are several restrictions on our investigation. First, the NHANES database is an observational study; weight affects BMI values based on a variety of factors, including physical examination, a child’s nutritional status, and additional weight bearing. Furthermore, we were unable to rule out the influence of additional confounders beyond the covariates in this article, so our findings might not accurately reflect the true situation. Second, due to limitations of the NHANES database, the study did not include the disease profiles of the patients, obesity brought on by medicine, congenital familial disorders, associated disorders, etc. SII and SIRI levels have been demonstrated to be correlated with a number of diseases, including hypertension, cardiovascular disease, insulin resistance, diabetes mellitus, and endocrine disorders. However, as there were so few children in this set of diseases, the relationships between BMI, SII, and SIRI and disease were not investigated further.

## 6 Conclusion

The findings suggest that SII and SIRI levels appears to be increasing as body mass index (BMI) increases, indicating that these parameters could be used as possible markers of inflammation in children that have been classified as overweight or obese.

## Data Availability

The datasets presented in this study can be found in online repositories. The names of the repository/repositories and accession number(s) can be found below: www.cdc.gov/nchs/nhanes/.
